# Abdominal Lump in a 32-year-old Male

**DOI:** 10.4103/1319-3767.77252

**Published:** 2011

**Authors:** Ankur Gadodia, Swati Thakur, Raju Sharma

**Affiliations:** Department of Radio-diagnosis, All India Institute of Medical Sciences, New Delhi, India

A 32-year-old man presented with vague pain and lump in the right hypochondrium and lumbar region for last six months. Physical examination revealed a mobile, nontender lump (6 × 6 cm) in hypogastrium. Patient had normocytic normochromic anemia and baseline serum creatinine was 2.1 to 2.9 mg%. Barium meal follow through (BMFT, Figure 1) and contrast enhanced computed tomography (CECT, Figure 2) were performed.

**Figure 1:**
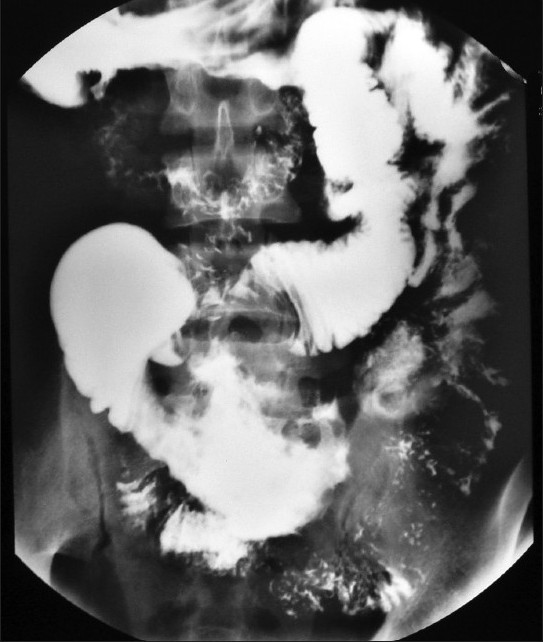
Barium meal follow through in a 32-year-old male with abdominal lump

**Figure 2:**
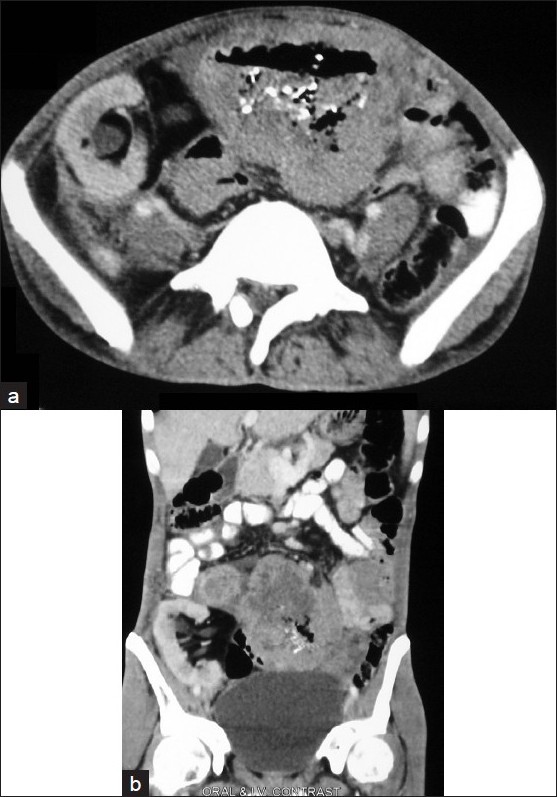
Contrast-enhanced axial (a) and coronal (b) computed tomography images in a 32-year-old male with abdominal lump

Patient underwent living related donor renal transplantation for treatment of end-stage renal disease caused by chronic interstitial nephritis seven years ago. Triple drug immunosuppression was initiated. The allograft functioned well and there were no rejection episodes for seven years.

## QUESTION

Q1. What is your diagnosis?

## ANSWER

## DIAGNOSIS

BMFT demonstrates a dilated segment of distal small bowel loops with areas of ulceration. Computed tomography (CT) of abdomen show irregular low-attenuating wall thickening with areas of ulceration involving the small bowel loops and aneurysmal dilatation of small bowel loop. Note the presence of transplanted kidney in right iliac fossa. Findings are suggestive of post transplant lymphoproliferative disorder (PTLD).

## DISCUSSION

Post transplant lymphoproliferative disorder (PTLD) is well-recognized, although relatively uncommon, and occurs in approximately 1% of renal allograft recipients.[1] In most cases, PTLD is associated with Epstein-Barr virus (EBV) infection of B cells, either as a consequence of reactivation of the virus posttransplantation or from primary posttransplantation EBV infection acquired from the donor.[2] While T-cell lymphoproliferative disorders not associated with EBV infection have also been documented after solid organ and bone marrow transplantation, the vast majority are B-cell proliferations.

A diagnosis of PTLD is made by having a high index of suspicion in the appropriate clinical setting; histopathological evidence of lymphoproliferation on tissue biopsy; and the presence of EBV DNA, RNA, or protein in tissue. Most cases of PTLD are observed in the first posttransplant year. The more intense the immunosuppression used, the higher the incidence of PTLD and the earlier it occurs. The cornerstone of successful treatment of PTLD is reduction or withdrawal of immunosuppression. Alimentary tract involvement by posttransplantation lymphoproliferative disorder is reported in 30% of transplant recipients with abdominal disease in a series.[3] The radiologic appearances are similar to that of non-Hodgkin’s lymphoma, with the exception that patients with PTLD have an increased propensity for ulceration and perforation of the alimentary tract. Involvement of the hollow abdominal viscera can manifest as circumferential mural infiltration with luminal excavation, a discrete eccentric mass with or without ulceration, or luminal narrowing.[4] Because alimentary tract disease has a propensity for extraluminal extension, CT is the imaging technique of choice to evaluate the extent of disease. Extranodal disease in PTLD is much more common than in lymphoma.

CT is a commonly performed study in symptomatic organ allograft recipients to evaluate complications related to transplantation. Recognition of the CT findings of PTLD is important because early diagnosis may result in an improved response to therapy, which often consists of only reducing the level of immunosuppression.[5]
